# Validation of a novel mHealth app to support foreign domestic workers in domiciliary eldercare

**DOI:** 10.3389/fdgth.2026.1862187

**Published:** 2026-07-27

**Authors:** Wei Lung Teo, Win Yee Cheong, Kim Wai Ang, Yi Ling Eileen Koh, Ngiap Chuan Tan

**Affiliations:** 1 Duke NUS Medical School, Singapore, Singapore; 2 SingHealth Polyclinics, Singapore, Singapore

**Keywords:** care-recipient, digital health, foreign domestic worker, geriatrics, informal caregiving, mobile-phone application

## Abstract

**Introduction:**

Many families in Singapore rely on Foreign Domestic Workers (FDWs) for domiciliary eldercare. However, FDWs often lack confidence and competence in providing basic healthcare to older adults, potentially compromising their health outcomes. This pilot randomized controlled trial (RCT) determined the feasibility, usability and effectiveness of E-CHEER, a prototype multilingual mobile-health application to upskill their domiciliary caregiving competency to senior care-recipients.

**Methods:**

Sixty FDW–employer dyads from two local public primary care clinics were recruited and randomized to intervention (*n* = 30; E-CHEER access) or control (*n* = 30; no app). Study feasibility outcomes included recruitment, retention, and adherence rate metrics. The System Usability Scale (SUS) determined the E-CHEER usability in the intervention group. FDWs' scores in caregiving knowledge (CK), video recordings of their blood pressure measurement (BPM), and ambulation supervision ability (AS) were remotely evaluated by independent clinician-assessor for effectiveness at baseline and follow-up at up to 12 weeks. Statistical analyses were conducted to evaluate intra- and intergroup outcomes.

**Results:**

Recruitment rate was 50.4% (60 dyads recruited from 119 approached). Among the 49 dyads who completed the study (retention 81.6%), almost all of them completed their assigned tasks. The intervention group showed significant intra-group improvements in BPM (*P* = 0.003), AS (*P* = 0.02), and CK (*P* = 0.002), and significant AS improvement (*P* = 0.03) compared to the control group. The mean SUS score was 62.6 (95% CI 58.9–66.3).

**Discussion:**

Implementation of E-CHEER app was feasible based on the response and retention rates and was effective in improving FDWs' supervision of their care-recipients during ambulation, but its usability needs enhancement.

## Introduction

The World Health Organization (WHO) projects that one in five individuals in the world would be aged 60 years or above, which will double to two billion senior people from the year 2019 to 2050 (1). Coupled with birth rates that consistently reach record lows year after year (2), this means that the level of care-giving burden on the younger population to support the healthcare needs of the older population will escalate significantly due to increasing disease burden, straining healthcare systems globally.

The rising burden of eldercare is similarly observed in Singapore, whose multi-ethnic Asian population has one of the highest average life expectancies in the world at 83 years, only trailing behind countries like Sweden and Japan (3). More healthcare resources are thus required to support their well-being as ageing invariably results in multiple co-morbidities (4). A suboptimal primary care response to cater to their healthcare needs will result in their adverse health outcomes and overload the local tertiary healthcare system.

Majority of seniors in Singapore (97%) age in place at home (5). Only a minority reside in nursing homes (2.5%) and even fewer live in assisted living facilities (0.1%) (6). A 2024 study also showed that 82.8% of local seniors prefer to age at home (7). In Asian communities like Singapore, children are traditionally expected to be the primary caregivers for their elderly parents who reside in the same households. However, such practice becomes difficult due to increasingly common dual-income earner work arrangements among the employed children. In addition, the shrinking family size due to declining fertility rate leads to fewer children taking direct care of their ageing parents. As a result, local working couples commonly hired contracted stay-in foreign domestic workers (FDWs) to look after the older and younger family members, aside from assisting with household chores. From 2015 to 2018, families which employed FDWs, largely from neighboring countries such as Indonesia, Myanmar and Philippines, increased from 12% to 14%, totaling about 90,000 households (8). English is the common language in Singapore for communication. The FDWs are expected to be literate in basic English before they can be brought in by professional agencies.

More than half of FDWs in Singapore were involved in providing healthcare needs to the older family member at home based on a recent local study (9). In 2021, the number of professionally trained live-in caregivers was only 1,600, a small proportion compared to the number of untrained FDWs providing home-based caregiving (10). Pre-employment training for FDWs by the employment agencies generally focuses on performance of household chores. These agencies are not mandated to provide caregiver training for FDWs to work in Singapore (11). Hence, FDWs managing older care-recipients at home usually learn to provide care on-the-job. The local study had reported FDWs' low confidence and competency in carrying out their caregiving role of supporting older care-recipients with specific healthcare needs, which could adversely impact on the health of the latter (9).

Falls are a major cause of morbidity and mortality among older adults in Singapore, with a crude incidence rate of 277.7 per 100,000 for adults aged 60 years and older (12). Recurrent falls occur in one third of local seniors aged 60 years and older, and 63% of these falls occur at home (12). FDWs therefore play an important role in mitigating fall risk by identifying and reducing environmental hazards such as slippery floors and tripping hazards, supporting older adults with physical and medical conditions such as muscle weakness, poor balance and gait, reminding them to use walking aids, and monitoring lifestyle and other factors such as side-effects from medication and improper footwear. Elevated or low blood pressure is also a known risk factor for falls, further highlighting the importance of equipping FDWs with the skills to monitor and manage the health of their care-recipients.

Ever since smartphones became ubiquitous, mobile health (mHealth) applications (app) have proliferated and are increasingly being deployed in healthcare, from helping patients to access healthcare services, to managing their long-term medical conditions (13–15). These apps have emerged as valuable tools for supporting informal caregivers, connecting them to community resources and improving their mental wellness. Nonetheless, apps which target FDWs who live in the same household but have different cultural, and linguistic background from their care recipients is lacking. The depth of understanding of the caregiving instructions from the employers among these largely native speaking FDWs due to language and sociocultural barriers remains unclear.

Such unmet needs create the opportunity for innovation to bridge the gap, including the development and testing of a novel linguistically inclusive app that is designed and engineered to upskill stay-in FDWs to provide basic healthcare to seniors in the households. The app pairs them with their employers, who will leverage on the mHealth intervention for closer engagement and supervision of their FDWs. The E-CHEER (Electronic Caregivers and Helpers in Enhancing Care for the Elderly) mobile app was specially designed to deliver multilingual, skill-focused learning modules (e.g., blood pressure measurement, ambulation supervision, healthy meal preparation) for the FDWs, incorporating assessment and feedback features from their employers.

The primary aim of this study was to assess the feasibility of implementing E-CHEER among FDWs to provide basic healthcare to their domiciliary care-recipients by examining the recruitment, retention and adherence rates to the study.

The secondary aims were to evaluate (1) the usability of the E-CHEER app based on scores from the System Usability Scale (SUS) questionnaire, and (2) the preliminary effectiveness of the app in improving the caregiving skill and knowledge amongst app users (intervention group) in comparison with the non-users (control group). Their skill performance was evaluated through employer-submitted video recordings of the FDWs performing blood pressure measurement (BPM), ambulation supervision (AS) of the care-recipients and the caregiving knowledge (CK) assessment scores.

## Materials and methods

### Development of E-CHEER mobile application

E-CHEER is a web-based mobile application developed by the lead investigator over 4 months. The interface and features are shown in Supplementary Figures S1, S2 in Supplementary File S1. Its features included:

1. Skill training module on blood pressure measurement and blood glucose monitoring procedures (Supplementary Figure S3 in Supplementary File S1)
2. Exercise supervision training module that teaches safe exercise conduct and ambulation supervision for care-recipients (Supplementary Figures S4, S5 in Supplementary File S1)
3. Meal preparation module that teaches healthy meal preparation for care-recipients (Supplementary Figures S6, S7 in Supplementary File S1)
4. Assessment module that enables employers to evaluate FDWs performing specific caregiving tasks (Supplementary Figure S8, S9 in Supplementary File S1)
5. Dual language support for FDWs, with the app interface and module content displayed in both English and the FDW's native language (Burmese for Myanmar FDWs and Bahasa Indonesia for Indonesian FDWs)
6. Caregiving knowledge quiz module with True/False questions for knowledge assessment
7. Real-time notification system to inform study participants when study tasks were due
8. A module enabling FDWs to view study team feedback following BPM assessments

Navigation within the app was facilitated through a hamburger menu (three-line icon) located at the top right of the interface (Supplementary Figure S2 in Supplementary File S1), through which users can access the different modules. The app featured a secure login mechanism which prevents unauthorized access (e.g., non-study participants, control group). Video resources were hosted on a secure approved server with download functionality disabled to prevent participants from sharing video resources between groups.

Training content was adapted from the face-to-face training workshop entitled CHEER (Caregivers and Helpers in Enhancing Care for the Elderly) in a primary care institution and meal preparation content from a national health portal, HeathHub.sg (16). Instructions were delivered via video, image, and text. Caregiving knowledge questions were based on content found in the various modules of E-CHEER. FDWs were permitted to revisit and repeat the modules without restriction throughout the study period.

The app interface screenshots are shown in Supplementary Material.

### Patient and public involvement in the study design

During the app developmental phase, the investigators consulted the institution Patient and Public Involvement (PPI) Partners to seek their views on FDWs' scope of work related to community-based eldercare and E-CHEER (17). They reviewed the research proposal, study design, recruitment approach, and the E-CHEER prototype app.

Their feedback led to several important refinements, including simplification of the English and equivalent language translation features to improve the FDWs' understanding of app content, and use of videos to deliver training content instead of solely text-based instructions. The instructions are color-coded, using red to emphasize vital tasks, and yellow to denote good-to-know tips.

### Trial site and study population

The pilot randomized control trial was conducted at two public primary healthcare clinics in eastern Singapore at Tampines and Pasir Ris, where a significant elderly population resides (estimated 98,000 as of 2025) (18).

A sample size of 30 participant per group is recommended by Browne RH for pilot testing of a novel intervention (19). The investigators planned to enrol 30 dyads per group, bringing the total planned recruitment to 60 dyads.

### Inclusion criteria

Participants comprised of dyads, each consisting of a FDWs and her employer. Both were required to have basic English proficiency and possess an internet-enabled mobile device. 97% of Singapore residents own a smartphone. The FDWs were screened to be actively taking care of senior members in the same household as their care-recipients; the latter had at least one diagnosed chronic disease being managed at either study site. The employers were capable and willing to use E-CHEER downloaded onto their mobile phone to record video of their FDW's performance in specific tasks and transmitted to the study team. FDWs who had been professionally trained to provide home-based eldercare were excluded from the study.

### Randomization

These dyads were randomised using a 1:1 ratio to either the intervention or control group using block randomisation with randomly varying block sizes of 4 and 6. Randomisation was performed in Stata with a computer-generated sequence, and allocations were placed in sequentially numbered opaque envelopes.

### Trial procedure

After obtaining their written consent and randomization, the dyads in both intervention and control groups completed baseline questionnaires. For the FDW, the questionnaire gathered data on their demographic and work profile and a Caregiving Knowledge (CK) quiz. The employer provided information on the demographic and clinical profile of the care-recipients. The dyads in the intervention group also rated the usability of E-CHEER independently using the System Usability Scale (SUS).

The dyads in the intervention group were given access to E-CHEER by the lead investigator, who inducted them to download the app to their mobile phone and the use of its functionalities. Next, the lead investigator inducted the employers to perform two assessment tasks: (1) recording and uploading a video of their FDW performing a blood pressure measurement on the care-recipient; (2) completing a checklist assessment of their FDW supervising the care-recipient during ambulation (Ambulation Supervision, AS) via E-CHEER. They re-assessed the FDWs by video recording their BPM and AS performance, which was then uploaded via the E-CHEER two to three weeks later, with an allowable extension of up to 12 weeks to accommodate scheduling or caregiving constraints.

### Definition of outcomes

#### Primary outcomes on feasibility of E-CHEER

1. Recruitment rate: the number of dyads successfully recruited into the study divided by the number of eligible dyads approached by the study team
2. Retention rate: the number of dyads who successfully completed the study divided by the number of recruited dyads
3. Adherence rate: the number of dyads who completed the required study procedures (video submissions, checklist assessments, caregiving knowledge quiz) within the stipulated study period (2–12 weeks post-enrolment), calculated among participants who completed the study

#### Secondary outcomes

1. E-CHEER Usability: SUS was used to assess the usability of the app (20, 21).
2. Comparison of basic healthcare competency of FDWs
  (a) Ambulation Supervision (AS): Employers used the 11-step checklist (Supplementary Figure S1 in Supplementary File S2) to observe and appraise their FDW while the latter supervised the care-recipient during ambulation activities.
  (b) Blood Pressure Measurement (BPM): An independent nurse clinician in the study team rated the performance of the FDW measuring the blood pressure of the care-recipient from the video using an 11-item procedural checklist (Supplementary Figure S2 in Supplementary File S2).
  (c) FDW Caregiving Knowledge (CK): The Caregiving Knowledge questionnaire was adopted from an earlier study (9) and comprised 11 true/false questions with a maximum possible score of 11 (Supplementary Figure S3 in Supplementary File S2). The questionnaire was translated into Bahasa Indonesia and Burmese to accommodate participants' language preferences. The questions were adapted from the E-CHEER training modules; the correct answers were available from the training materials.
  (d) FDW Caregiving Confidence: The assessment comprises 5 questions measuring FDWs' confidence in caregiving tasks (Supplementary Figure S4 in Supplementary File S2). The scores range from “Not confident” (0 point), “A little confident” (1 point), “Moderately confident” (2 points), to “Very confident” (3 points). The mean score was used to measure the level of caregiving confidence.

### Blinding

The assessor was blinded to participants’ study identification and group allocation throughout the evaluation process. The blinding was achieved by ensuring videos sent to the assessor was labelled only with a random number. These random numbers were mapped to the participants' study identity, which was known only to the co-investigator who recruited the participants.

### Statistical methods

Statistical analysis was conducted using R software (V.4.4.2). Descriptive statistics were calculated for feasibility outcomes as well as FDW participant characteristics, caregiving demographics. For the caregiving competence outcomes, within-group changes from baseline to follow-up were analyzed using Wilcoxon signed-rank tests, and between-group differences in change scores were evaluated using Mann–Whitney *U*-tests. Effect sizes were calculated as r using Rank Biserial Correlation for both within-group and between-group comparisons. Statistical significance was set at *P* < 0.05.

### Ethics board approval

The study protocol adhered to the Declaration of Helsinki. The trial obtained ethical approval from the SingHealth Centralized Institutional Review Board (CIRB Ref: 2024–3655).

## Results

**Figure 1:**
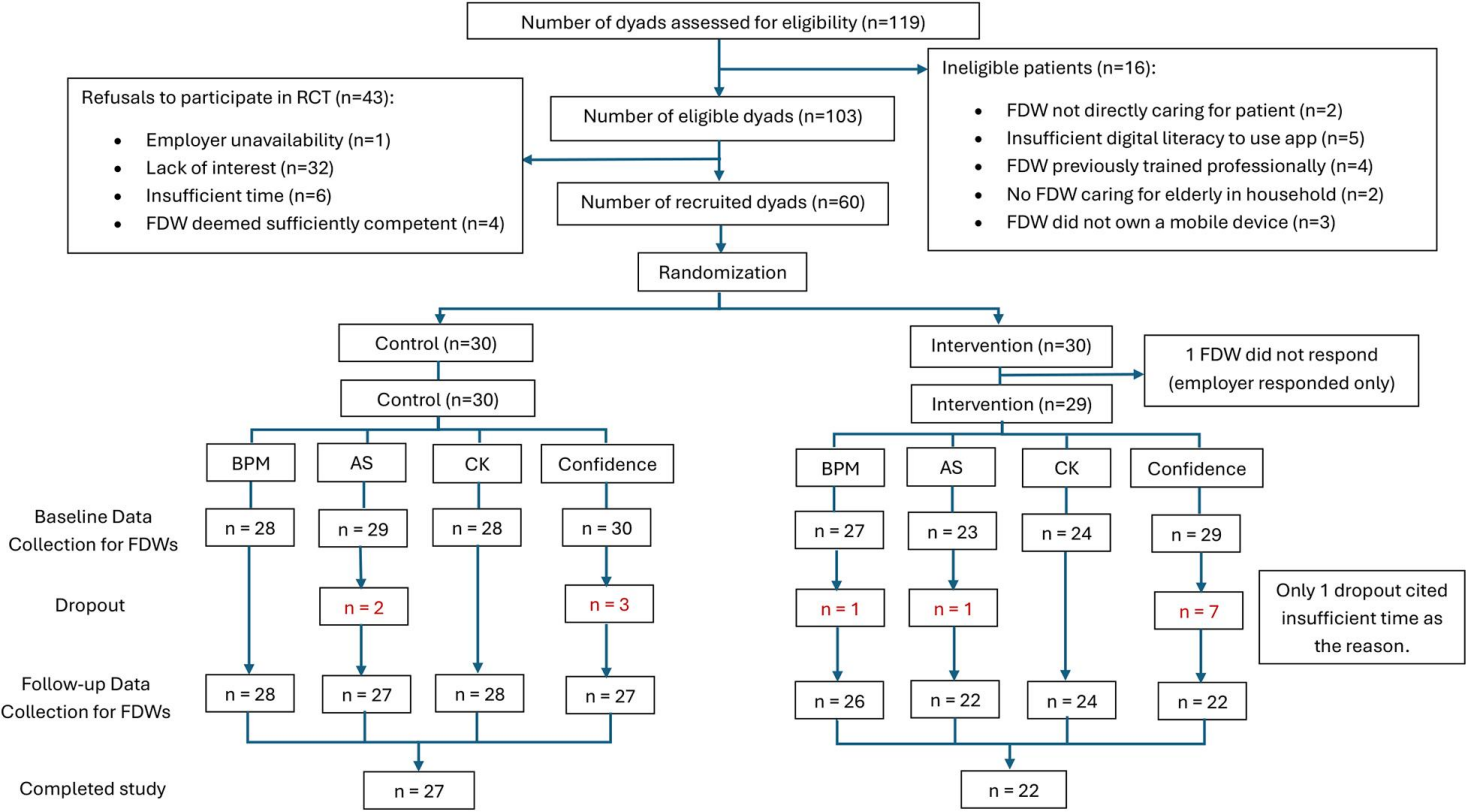
CONSORT diagram for the E-CHEER RCT.

The dyads' recruitment and retention rates are illustrated in the CONSORT diagram (Figure 1).

### Primary outcomes

A total of 119 FDW-employer dyads were approached for trial recruitment over 33 weeks from 11 December 2024 to 26 July 2025. Eventually, 60 dyads were successfully recruited, representing a recruitment rate of 50.4%.

The common reasons for rejecting trial participation were employer unavailability or their self-reported lack of interest at the study sites. Other reasons included FDWs who were not directly looking after the patients and/or were not digitally savvy to use or download E-CHEER to their mobile phone or were previously trained professionally to provide healthcare support to senior adults.

### Demographic profile of the FDWs

Tables 1, 2 present the baseline demographic and caregiving profiles of FDWs in the control and intervention groups respectively.

**Table 1:** Baseline characteristics of the FDWs in intervention and control groups.

Characteristics	Total, *n* (%)	Control, *n* (%)	Intervention, *n* (%)
	(*N* = 59)	(*N* = 30)	(*N* = 29)
Age (Years) (Median (IQR))	36.00 (30.00–41.00)	33.50 (28.00–40.75)	36.00 (30.00–42.00)
Nationality			
Myanmar	26 (44.1)	17 (56.7)	9 (30.0)
Indonesia	23 (39.0)	9 (30.0)	14 (48.3)
Philippines	9 (15.3)	3 (10.0)	6 (20.7)
India	1 (1.7)	1 (3.3)	0 (0.0)
Marital Status			
Married	23 (39.0)	12 (40.0)	11 (37.9)
Single/ separated/ divorced/ widowed	36 (61.0)	18 (60.0)	18 (62.1)
Highest educational level attained			
Primary	9 (15.3)	5 (16.7)	4 (13.8)
Junior Secondary	9 (15.3)	5 (16.7)	4 (13.8)
Senior Secondary	26 (44.1)	12 (40.0)	14 (48.3)
Tertiary	15 (25.4)	8 (26.7)	7 (24.1)
Number of years as a domestic helper (Median (IQR))	5.00 (2.00–9.00)	4.50 (1.25–8.00)	7.00 (3.00–9.00)
Previous experience as caregiver for elderly			
Yes	27 (45.8)	15 (50.0)	12 (41.4)
No	32 (54.2)	15 (50.0)	17 (58.6)
Received caregiver training for elderly			
Yes	7 (11.9)	3 (10.0)	4 (13.8)
No	52 (88.1)	27 (90.0)	25 (86.2)
Duration looking after the care recipient			
Less than 1 year	14 (23.7)	6 (20.0)	8 (27.6)
1–2 years	23 (39.0)	14 (46.7)	9 (31.0)
>2 years	22 (37.3)	10 (33.3)	12 (41.4)
Main language used to care for recipients			
English	38 (64.4)	17 (56.7)	21 (72.4)
Chinese/ Chinese dialects	10 (17.0)	6 (20.0)	4 (13.8)
Malay	9 (15.2)	5 (16.7)	4 (13.8)
Others	2 (3.4)	2 (6.7)	0 (0.0)

**Table 2:** Caregiving demographics of the FDWs in intervention and control groups.

Caregiving demographics	Total, *n* (%)	Control, *n* (%)	Intervention, *n* (%)
	(*N* = 59)	(*N* = 30)	(*N* = 29)
Caregiving tasks for care recipient^a^			
Prepare main meals	49 (83.1)	23 (76.7)	26 (89.7)
Ensure safety during exercise	38 (64.4)	21 (70.0)	17 (58.6)
Ensure correct dosage of medications	35 (59.3)	19 (63.3)	16 (55.2)
Measure home blood pressure	35 (59.3)	23 (76.7)	12 (41.4)
Measure home blood glucose	9 (15.3)	6 (20.0)	3 (10.3)
Assist the care recipient with			
Eating (e.g., cut food into small pieces)	32 (54.2)	14 (46.7)	18 (62.1)
Bathing (e.g., dress/undress, prepare cloth)	28 (47.5)	20 (66.7)	8 (27.6)
Dressing (e.g., undress or put on cloth, buttoning)	26 (44.1)	18 (60.0)	8 (27.6)
Toileting (e.g., bring to toilet, undress or wipe)	25 (42.4)	15 (50.0)	10 (34.5)
Transferring (e.g., from bed to chair and back)	24 (40.7)	16 (53.3)	8 (27.6)
Continence/diaper (e.g., change diaper)	19 (32.2)	14 (46.7)	5 (17.2)
Time spent on caregiving tasks			
A little	17 (28.8)	6 (20.0)	11 (37.9)
Moderate	26 (44.1)	14 (46.7)	12 (41.4)
Most of the time	16 (27.1)	10 (33.3)	6 (20.7)
Number of hours spent on caregiving per day (Median (IQR))	5.00 (2.50–10.00)	5.00 (3.00–8.00)	6.00 (2.00–10.00)
Bring care recipient for medical follow-up			
Yes	49 (83.1)	25 (83.3)	24 (82.8)
No	10 (16.9)	5 (16.7)	5 (17.2)
Share caregiving tasks with family members			
Yes	24 (40.7)	11 (36.7)	13 (44.8)
No	35 (59.3)	19 (63.3)	16 (55.2)
Other non-caregiving domestic tasks^a^			
Any household chores	57 (96.6)	29 (96.7)	28 (96.6)
Taking care of children	6 (10.2)	2 (6.7)	4 (13.8)
Taking care of another elderly person	6 (10.2)	2 (6.7)	4 (13.8)
Taking care of pets	2 (3.4)	2 (6.7)	0 (0.00)
Washing car	16 (27.1)	9 (30)	7 (24.1)
No need to do other domestic tasks	1 (1.7)	1 (3.3)	0 (0.00)

a Some FWDs may do more than one type of caregiving tasks, and other non-caregiving domestic tasks.

Table 1 shows that majority of FDWs (83.1%) were from Indonesia and Myanmar, and most (76.3%) of them were educated up to secondary level. While most (88.1%) had received formal caregiver training for the elderly, about half of them had prior actual caregiving experience. Most FDWs (76.3%) had been caring for their current care recipient for at least 1 year. The primary language used for caregiving was English (64.4%).

Table 2 shows that FDWs reported multi-tasking for their care-recipients, including main meal preparation (83.1%) and ensuring safety during exercise (64.4%), with over half responsible for medication dosage monitoring (59.3%) and blood pressure measurement (59.3%). They also assisted in feeding the care recipients, (54.2%), followed by bathing (47.5%) and dressing (44.1%).

Over a quarter of FDWs (27.1%) reported spending most time on caregiving, with an average of 6.5 h daily, yet the over half (59.3%) had little support from family members in sharing caregiving responsibilities. Nearly all FDWs (96.6%) performed additional household chores beyond caregiving, and 83.1% had to accompany care recipients for their medical appointments.

The study achieved an overall retention rate of 81.6%, with 49 out of 60 recruited dyads completing all study procedures (Table 3). The control group demonstrated higher retention (90%, 3 dropouts) compared to the intervention group (73.3%, 8 dropouts). Most participants did not provide specific reasons for withdrawal. Few of them cited inability to find time for study procedures.

**Table 3:** Retention and adherence rates by caregiving competency outcome measure.

Outcomes	Baseline, *n*			Follow-up, *n*			Participant Completion Rate (%)^a^			Adherence Rate, *n* (%)^b^	
	Total	Control	Intervention	Total	Control	Intervention	Total	Control	Intervention	Adherent, *n* (%)	Non-adherent, *n* (%)
BPM	55	28	27	54	28	26	90.0	93.3	86.7	48 (98.0)	1 (2.0)
AS	52	29	23	49	27	22	81.7	90.0	73.3	49 (100.0)	0 (0.0)
CK	52	28	24	52	28	24	86.7	93.3	80.0	47 (96.0)	2 (4.0)
Confidence	59	30	29	49	27	22	81.7	90.0	73.3	49 (100.0)	0 (0.0)

a Retention rate is calculated as (follow-up n/59) × 100 per outcome, where 59 represents dyads who provided baseline data (control *n* = 30, intervention *n* = 29).

b Adherence rate is calculated as (n/49) × 100 per outcome, where 49 represents dyads who completed follow-up data for all outcomes.

Table 4 demonstrates significant intra-group improvements in the intervention group from baseline to follow-up for BPM (*P* = 0.003), AS (*P* = 0.02), and Confidence (*P* = 0.002), with large within-group effect sizes for BPM (*r* = 0.73), AS (*r* = 0.63) and Confidence (*r* = 0.80). The intervention group showed significant improvement in score only for AS (*P* = 0.03) with a moderate effect size (*r* = 0.32).

**Table 4:** Results of analysis of BPM, aS, CK and confidence of FDWs within- and between-group at baseline vs. follow-up.

Outcomes	Control					Intervention							Rank-Biserial Correlation r		
	*n*	Baseline	Follow-up	Change (*Δ*)	*p*-value (*Δ*C)	*n*	Baseline	Follow-up	Change (*Δ*)	*p*-value (*Δ*I)	95% CI (*Δ*I—*Δ*C)	*p*-value (*Δ*I vs. *Δ*C)	Within		Between
		Median [IQR]	Median [IQR]	Median [IQR]			Median [IQR]	Median [IQR]	Median [IQR]				Control	Intervention	
BPM	28	8.00 (6.00, 10.00)	9.00 (7.00, 10.00)	0.50 (−1.00, 1.25)	0.16	26	8.50 (7.00, 9.00)	9.00 (8.25, 10.00)	1.00 (0.25, 2.00)	0.003	(0.00, 2.00)	0.20	0.32	0.73	0.20
AS	27	6.00 (4.50, 10.00)	7.00 (4.50, 11.00)	0.00 (0.00, 1.00)	0.12	22	4.00 (4.00, 6.75)	6.00 (5.00, 7.75)	1.00 (0.00, 2.75)	0.02	(0.00, 2.00)	0.03	0.53	0.63	0.36
CK	28	8.00 (8.00, 9.00)	9.00 (7.75, 9.00)	0.00 (0.00–1.00)	0.18	24	9.00 (8.00, 10.00)	9.00 (8.00, 10.00)	1.00 (−0.25, 1.00)	0.27	(0.00, 1.00)	0.47	0.38	0.28	0.12
Confidence	27	2.40 (1.60, 2.70)	2.40 (2.10, 3.00)	0.00 (0.00, 0.50)	0.06	22	1.70 (1.05, 2.00)	2.40 (2.05, 2.40)	0.40 (0.00, 1.20)	0.002	(0.00, 0.60)	0.09	0.54	0.80	0.30

BPM, Blood Pressure Measurement; AS, Ambulation Supervision; CK, Caregiving Knowledge; C, Control; I, Intervention; BPM, AS, CK scores are out of 11. Confidence scores are out of 3 (mean).

Participants who dropped out were excluded from analyses. Baseline characteristics of retained participants did not differ significantly from the full sample (*p* > 0.05).

**Figure 2:**
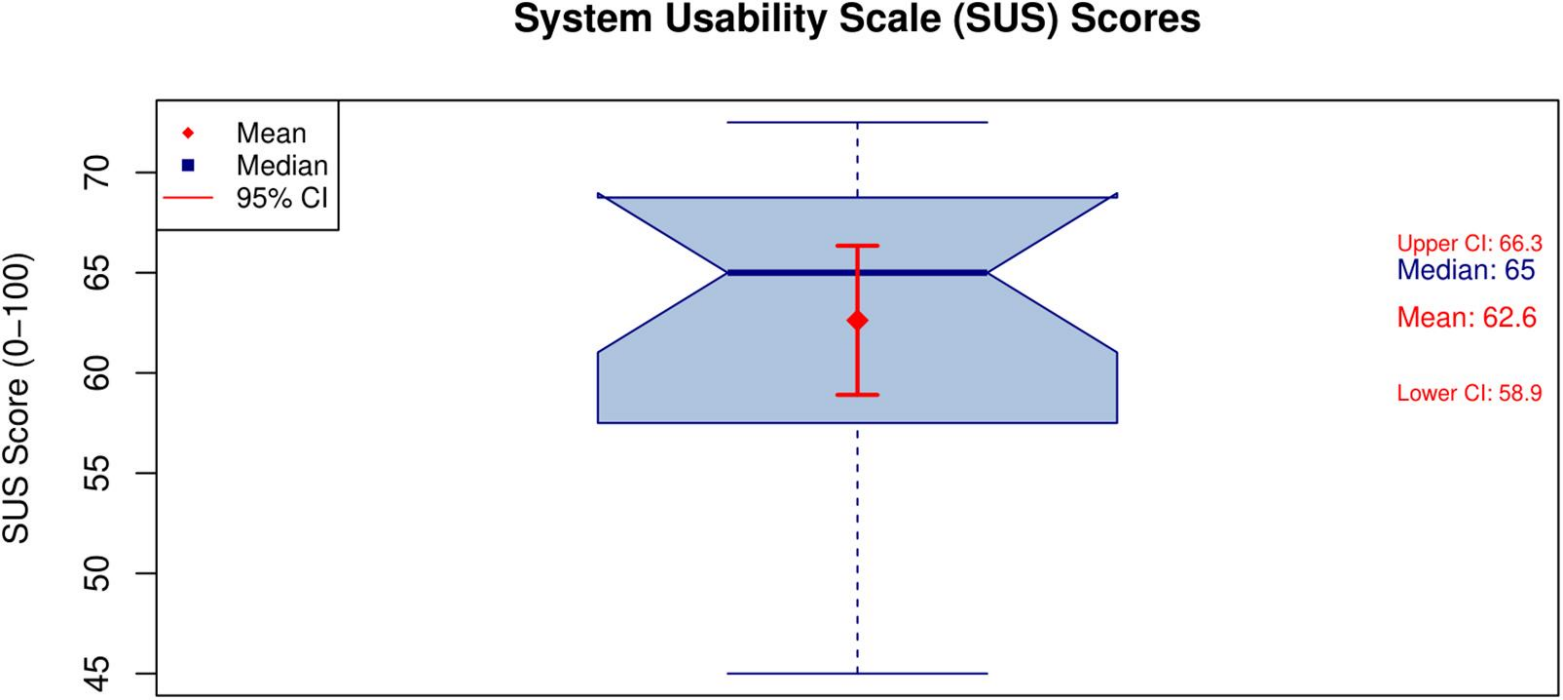
System usability scale (SUS) scores for E-CHEER.

The usability assessment revealed a mean SUS score of 62.6 (95% CI 58.9–66.3), which was within the acceptable range for usability but below the established benchmark of 68 (Figure 2).

## Discussion

This pilot RCT demonstrated the feasibility of implementing a novel mobile-phone application-based digital intervention involving an employer-FDW dyad to enhance basic geriatric home care. The intervention is deliberately designed to couple the employer and the FDW: one is likely the kin who is working to provide for the family and the other has close interaction with the senior adult most of the time. The relatively good retention and completion rates suggest that the dyad can work closely as vital partners to ensure that their senior adults live and age well in their home. The employers' willingness to voluntarily submit video assessments of their respective FDWs without any incentive was encouraging. Family support for older care recipients is essential for their optimal care at home, including taking a proactive role to equip the FDWs with basic clinical skills. Employers can consider using E-CHEER as an ancillary and accessible remote basic clinical training tool for their FDWs if the next iteration of the app design is implemented in the community.

Nonetheless, the modest recruitment rate (50.4%) reflects trial protocol challenges in enrolling both the FDW and her employer together in the same setting, which is more difficult than enrolling single eligible participants in most studies. The employer might not accompany their older relative to the study site during their medical review due to work or other commitments and missed the opportunity to join the trial. Even when present, employers often cited time constraints and perceived mental burden to take part in the trial. A few employers expressed limited trust in their FDWs or hesitancy to allow them to use their mobile devices for research-related activities. These factors collectively contributed to slower recruitment and required additional engagement efforts by the study team. Such barriers in engaging the employers can be addressed using other media platform to introduce and induct them to the use of E-CHEER and to demonstrate its functions and modus operandi.

The trial was implemented as a pilot to gain insight into the acceptability and usability of a novel mHealth application by the target users. No precedent digital intervention has been tested to upskill the FDWs aside from more resource intensive in-person training by professional agency. Nevertheless, the mean SUS score of 62.6 suggests further app refinement is needed to enhance its usability, with the goal of attaining or exceeding the benchmark score of 68. Amongst FDWs who rated the app unfavorably on the SUS, they cited the intensive induction and navigation challenges in the app, and the need for on-site technical support, arising from specific questions on the scale (“I needed to learn a lot of things before I could get going with this system”, “I think that I would need the support of a technical person to be able to use this system.” and “I found the system unnecessarily complex.”)

Aside from input from the PPI members, engaging the FDWs and their employers to co-design to improve the E-CHEER app is another strategy. The E-CHEER 2.0 app will incorporate features such as target user testing, multilingual interface simplification, and design adjustments informed by in-depth qualitative feedback to raise its overall usability and satisfaction levels. The investigators will also explore more favorable methods to expedite the user induction and timely backend technical support embedded in the next app prototype. Peer support among FDWs either in-person during clinic visit in group education talk or e-chat group function incorporated in the app are potential solutions.

The FDWs in the intervention group showed significant improvements in performing BPM, AS, and care confidence between baseline and follow-up. These improvements were accompanied by large effect sizes (rank-biserial correlation *r* = 0.73, 0.63, and 0.80 respectively), indicating substantial gains in practical caregiving competencies following exposure to the E-CHEER intervention. These findings collectively suggest that structured, linguistically compatible e-learning method via an app can improve both technical caregiving skills among FDWs and boost their confidence in providing care to their senior care-recipients. However, the score in the care knowledge did not show significant change among these FDWs. Half of them had prior experience in eldercare, which provided opportunity for them to gain relevant knowledge on-the-job. Hence, the E-CHEER may be more helpful to raise the literacy of novice FDWs without exposure to domiciliary geriatric care.

In contrast, the FDWs in the control group exhibited small, non-significant changes across major outcomes, likely reflecting Hawthorne effect (22) and possibly natural learning or practice effects associated with repeated assessment. Informal knowledge exchange between FDWs and employers during follow-up assessments could have confounded the outcomes in both groups, when observational learning and task guidance occurred in real-world setting.

Between-group analyses revealed a significant difference in ambulation supervision (*P* = 0.03) among the FDWs in intervention group compared with controls. No statistically significant inter-group differences were observed for BPM, caregiving knowledge, or confidence outcomes. The preliminary effectiveness indicates that minimally E-CHEER inducted the FDW to better supervise the older adults in the same households to reduce their fall risk during ambulation. While BPM did not show significant between-group differences in this pilot study, the BPM module may also contribute to fall risk reduction by enabling FDWs to identify hypotension or highly elevated blood pressure, which requires further evaluation.

The non-significant differences for the between-group analyses for BPM (*P* = 0.20) and caregiving knowledge(*P* = 0.47) are not unexpected as this is a pilot study with a small sample size, hence statistically significant differences might not have been detected. Additionally, caregiving knowledge may decay over time as participants might not have fully retained the information learned from the modules during the follow-up assessment. Future definitive trials with larger sample size, longer follow-up period and serial intervention to sustain learning by the FDWs would be better powered to detect differences in these outcomes.

E-CHEER is likely to be the first digital intervention in the world that aims to train stay-in migrant domestic workers to provide basic healthcare to senior adults in a developed community. The preliminary effect size from this pilot trial will enable the design of an adequately powered randomized controlled trial based on a revised app to attest its effectiveness in upskilling the novice FDWs in domiciliary eldercare.

Employers were inducted to video record their FDWs' carrying out the assigned tasks such as BPM. However, such videos recorded by the lay employers might incidentally capture the facial or other identifiable features of the FDWs, but the videos were deleted by the study team after the trial to ensure privacy of the FDWs. The nurse clinician who assessed the video independently had no interaction with the FDWs, whose personal data was masked using study identification number. The trial was also limited by the short follow-up period of up to 12 weeks, which did not allow evaluation of long-term retention of caregiving skills or sustained behavior change. The absence of qualitative feedback restricted deeper exploration of user experience and contextual barriers to app use.

## Conclusion

This pilot randomized controlled trial showed E-CHEER as a feasible digital training tool for migrant domestic workers involved in home-based eldercare from the satisfactory recruitment, retention, and adherence rates to the study procedures. The E-CHEER showed preliminary effectiveness in enabling FDWs to supervise the care-recipients in ambulation and enhancing their eldercare confidence. A significant between-group improvement was observed for ambulation supervision, suggesting that the intervention may potentially value-add to fall prevention among the older adults.

## Data Availability

The raw data supporting the conclusions of this article will be made available by the authors, without undue reservation.
