# Supplementary material for: Validation of a novel mHealth app to support foreign domestic workers in domiciliary eldercare
Source: Front Digit Health. 2026 Jul 27;8:1862187. doi: 10.3389/fdgth.2026.1862187 (PMC13454892; doi:10.3389/fdgth.2026.1862187)
Supplement: Supplementary file 1 [file Datasheet1.docx]

## Supplementary Material

This document provides screenshots of the E-CHEER mobile application used in the study to illustrate the user interface. The following screenshots were taken from the test version of E-CHEER. The application is still in development and hence may be subject to changes.

Figure S1: Main menu of E-CHEER

Figure S2: Features of E-CHEER

Figure S3: Skill module showing the blood pressure skill

Figure S4: Exercise module dropdown menu displaying available exercises for FDWs

Figure S5: Exercise module showing ambulation exercise content

Figure S6: Meal preparation module introduction showing an interactive healthy plate image

Figure S7: Meal preparation module: "Brown Rice & Wholemeal Bread" details from the healthy plate

Figure S8: Assessment module for employer evaluation of FDW ambulation competency

Figure S9: Assessment module submission screen
