# Supplementary material for: Validation of a novel mHealth app to support foreign domestic workers in domiciliary eldercare
Source: Front Digit Health. 2026 Jul 27;8:1862187. doi: 10.3389/fdgth.2026.1862187 (PMC13454892; doi:10.3389/fdgth.2026.1862187)
Supplement: Supplementary file 2 [file Datasheet2.docx]

Figure S1: Ambulation Supervision Checklist

|  | **Pre-ambulation Assessment:** |
| --- | --- |
| 1 | Checks if care recipient is experiencing dizziness or pain before commencing ambulation |
| 2 | Checks and prepares appropriate walking aid (if required) |
| 3 | Assists care recipient to sit at bedside and allows time for adjustment |
|  | **During Ambulation:** |
| 4 | Stands beside care recipient and assists them to stand up slowly |
| 5 | Conducts warm-up exercises (walking on the spot) and checks for dizziness before commencing walk |
| 6 | Walks alongside or near the care recipient throughout the ambulation session |
| 7 | Provides physical support to care recipient only when necessary |
| 8 | Identifies and removes environmental hazards along the walking path |
|  | **Post-ambulation Assessment:** |
| 9 | Help care recipient sit back down slowly and safely |
| 10 | Checks if care recipient is experiencing pain, breathing difficulties, or dizziness following ambulation |
| 11 | Uses proper body mechanics (squatting rather than bending back) when assisting care recipient during ambulation |

*Source: Adapted with permission from reference 1*

Figure S2: Blood Pressure Management Checklist

|  | **Assessment:** |
| --- | --- |
| 1 | Checks if care recipient has consumed coffee or smoked within the last 30 minutes |
| 2 | Ensures care recipient has been seated for at least 5 minutes prior to measurement |
| 3 | Verifies that the blood pressure machine has sufficient battery power |
| 4 | Checks care recipient's arm for any injuries or contraindications |
| 5 | Ensures care recipient's palm is facing upward |
| 6 | Rolls up sleeves appropriately (if not sleeveless) |
| 7 | Positions cuff tubing/arrow facing upward when securing to care recipient's arm |
| 8 | Places cuff just above the elbow (avoiding placement too high near shoulders) |
| 9 | Presses the blood pressure machine button to initiate measurement |
| 10 | Correctly identifies all three readings (systolic pressure, diastolic pressure, and pulse rate) |
| 11 | Records all three measurements in appropriate documentation (e.g., blood pressure diary) |
| 12 | Removes cuff and turns off blood pressure machine |

Source: Adapted with permission from references 2-3

Figure S3: Caregiving Knowledge Quiz

| Question | Statement | Correct Answer |
| --- | --- | --- |
| 1 | Healthier cooking methods include using less oil, fat and salt | True |
| 2 | I should choose products with healthier choice symbol or with A/B Nutri-grade label | True |
| 3 | Soupy dishes are less healthy than food with gravy | False |
| 4 | According to the Healthy Plate, older people should have 6 servings of fruit and 6 servings of vegetables a day | False |
| 5 | According to the Healthy Plate, eating 4 slices of wholemeal bread = 1 serving wholemeal bread | False |
| 6 | Exercise can help to lose weight and improve sugar/blood pressure control | True |
| 7 | It is important to check with doctor/nurse to decide what exercise is suitable and safe | True |
| 8 | Exercise is not important for elderly | False |
| 9 | Home blood pressure should be checked in the morning only | False |
| 10 | Blood pressure may be higher at the clinic than at home due to factors such as lack of sleep or anxiety at the clinic | True |
| 11 | Checking blood sugar at home tells you if the blood sugar level is too high or too low | True |

Source: Adapted with permission from references 4-5

Figure S4: Task confidence questions

|  | **Question** |
| --- | --- |
| 1 | How confident are you in preparation of special diet for care recipient? |
| 2 | How confident are you in preparation/supervision of medications for care recipient? |
| 3 | How confident are you in supervision of suitable exercise (e.g. walking) for care recipient? |
| 4 | How confident are you in using monitoring devices set, glucometer set) for care recipient? |
| 5 | How confident are you in changing diapers for the care recipient? |

*Source: Adapted with permission from reference 6*

Supplementary References

1. Singapore General Hospital. Esther_Let’s Get Moving [Internet]. 2023 [cited 2026 Jul 7]. Available from: <https://www.youtube.com/watch?v=QJBNssNcMgE>
2. WHO Technical Specifications for Automated Non-Invasive Blood Pressure Measuring Devices with Cuff. 1st ed. Geneva: World Health Organization; 2020. 1
3. CDC. High Blood Pressure [Internet]. 2026 [cited 2026 Jul 7]. Measuring Your Blood Pressure. Available from: <https://www.cdc.gov/high-blood-pressure/measure/index.html>
4. SingHealth Polyclinics. Caregiving Training for Foreign Domestic Workers [unpublished PowerPoint slides]. Singapore: SingHealth Polyclinics; 2024.
5. HealthHub. Nutritious Foods For A Healthy Diet [Internet]. Singapore: HealthHub; [date unknown] [cited 2026 Apr 14]. Available from: <https://www.healthhub.sg/programmes/nutrition-hub/eat-more>
6. Ang KW, Koh YLE, Wang XF, Yusoff AM, Tan AM, Poh XYC, et al. Pilot cross-sectional study of foreign domestic workers supporting the basic healthcare needs of older persons in the community. BMJ Open. 2022 Mar;12(3):e051877. doi:[10.1136/bmjopen-2021-051877](https://ddei5-0-ctp.trendmicro.com:443/wis/clicktime/v1/query?url=https%3a%2f%2fdoi.org%2f10.1136%2fbmjopen%2d2021%2d051877&umid=77DB310A-55FC-8306-A5F9-E586D76C221D&auth=ce6a3f6d14c1e3e3f878a8a607dd7ee2cb794b08-5affbc37bf636887dce16d16cca503b667e74815)
